# Effects of a beetroot juice with high neobetanin content on the early-phase insulin response in healthy volunteers

**DOI:** 10.1017/jns.2014.7

**Published:** 2014-04-30

**Authors:** Peter C. Wootton-Beard, Kirsten Brandt, David Fell, Sarah Warner, Lisa Ryan

**Affiliations:** 1 Functional Food Centre, Oxford Brookes University, Gipsy Lane, Oxford OX3 0BP, UK; 2 Human Nutrition Research Centre, School of Agriculture and Rural Development, Newcastle University, Newcastle upon Tyne NE1 7RU, UK; 3 Cell Systems Modelling Group, Oxford Brookes University, Gipsy Lane, Oxford OX3 0BP, UK; 4 Department of Nutrition and Dietetics, Monash University, Faculty of Medicine, Nursing and Health Sciences, 264 Ferntree Gully Road, Vic 3168, Australia

**Keywords:** Postprandial glycaemia, Phytochemicals, Betalains, BEET, beetroot beverage, CHO, carbohydrate, GLUC, glucose beverage, iAUC, incremental AUC, MCON, matched control beverage, sAUC, segmental AUC

## Abstract

Produce rich in phytochemicals may alter postprandial glucose and insulin responses by interacting with the pathways that regulate glucose uptake and insulin secretion in humans. The aims of the present study were to assess the phytochemical constituents of red beetroot juice and to measure the postprandial glucose and insulin responses elicited by either 225 ml beetroot juice (BEET), a control beverage matched for macronutrient content (MCON) or a glucose beverage in healthy adults. Beetroot juice was a particularly rich source of betalain degradation compounds. The orange/yellow pigment neobetanin was measured in particularly high quantities (providing 1·3 g in the 225 ml). A total of sixteen healthy individuals were recruited, and consumed the test meals in a controlled single-blind cross-over design. Results revealed a significant lowering of the postprandial insulin response in the early phase (0–60 min) (*P* < 0·05) and a significantly lower glucose response in the 0–30 min phase (*P* < 0·05) in the BEET treatment compared with MCON. Betalains, polyphenols and dietary nitrate found in the beetroot juice may each contribute to the observed differences in the postprandial insulin concentration.

Polyphenols including flavonoids, phenolic acids, proanthocyanidins and tannins have been suggested to be able to modify postprandial glycaemia^(^^1^^,^^2^^)^. Polyphenols may alter glycaemia by inhibiting carbohydrate (CHO) digestion, reducing CHO absorption in the intestines, stimulation of insulin release from pancreatic β-cells, modulation of hepatic glucose output, activation of insulin receptors, or modulation of glucose uptake in insulin-sensitive cells^(^^3^^)^. Isoflavonoids (soya), condensed tannins such as epigallocatechin-3-gallate from tea, phenolic acids (coffee), resveratrol (grapes), apple flavonoids, terpenoids (herbs), as well as cranberry, strawberry and blueberry anthocyanin metabolites have been studied for their anti-hyperglycaemic effects (for a review, see Hanhineva *et al.*^(^^2^^)^). The development of dietary components that positively influence postprandial glycaemia is of upmost importance because of their potential to reduce the impact of type 2 diabetes. The global incidence of type 2 diabetes is predicted to reach 360 million cases by the year 2030^(^^4^^)^, clearly articulating the need for investigations into the anti-hyperglycaemic effects of plant foods. The release and uptake of insulin are central to the absorption and transport of glucose from an ingested meal. Meals containing a large amount of CHO elicit a proportionate rise in plasma glucose that stimulates a rapid rise in blood insulin, termed insulinaemia. Repeated bouts of hyperglycaemia and hyperinsulinaemia may result in insulin resistance or transient hypoglycaemia owing to the rapid rise and fall of blood glucose. Insulin resistance is central to the development of type 2 diabetes and is one pillar of the metabolic syndrome, diseases that place a high economic burden on global societies^(^^5^^)^. Polyphenols and related compounds have been described to reduce both postprandial hyperglycaemia and prevent reactive hyperinsulinaemia by reducing the digestion, absorption and transport of glucose^(^^6^^–^^9^^)^.

Beetroot juice has received attention in the scientific literature recently, particularly due to its nitrate content^(^^10^^)^. Dietary nitrate is converted to nitrite by anaerobic bacteria in the saliva and subsequently to NO in the stomach, which complements endogenous NO production from l-arginine^(^^11^^)^. Excess nitrate has been linked to gastrointestinal/gastro-oesophageal cancers in adults^(^^12^^)^ and to methaemoglobinaemia in infants^(^^13^^)^. Nitrate is consumed in the diet primarily from processed meats, fish and from vegetable sources. The beneficial effects of nitrate from plant sources have been reviewed in detail^(^^14^^)^ and, as such, consumption of nitrate-rich vegetables is encouraged, whilst a reduction in processed meat is advised to ameliorate potential negative consequences. However, nitrate is only one component of the traditional root vegetable beetroot, and other investigations have sought to determine its phytochemical composition^(^^15^^–^^17^^)^. Beetroot contains a number of compounds including phenolic acids, flavonoids and betalains^(^^15^^)^, and beetroot juice has a high total antioxidant capacity and total polyphenol content as measured by the Folin–Ciocalteu method^(^^16^^,^^17^^)^. Furthermore, in terms of available CHO, beetroot is composed of approximately 90 % sucrose. Beetroot juice is therefore an interesting food model to investigate any influence of its bioactive components on the glycaemic response, either by direct inhibition of glucose uptake or by indirect action affecting insulin sensitivity. The aims of the present study were to identify and measure the phytochemical constituents of a commercial beetroot juice product, and to quantify and compare the glucose and insulin responses to a total available CHO intake of 50 g, delivered as either 225 ml of beetroot juice, or 225 ml of a control beverage matched for macronutrient composition or a positive control of 225 ml of a glucose-only beverage. Beetroot juice and its components have not been studied in this context before. Their major components, betalains, bear certain structural similarities to anthocyanins that have been shown to elicit alterations in blood glucose and insulin responses. It is, therefore, important to consider the potential impact of betalains as beetroot is widely available, easy to cultivate, pleasant tasting and relatively cheap for manufacturers and consumers alike.

## Experimental methods

### Determination of nitrate, nitrite, betalain and phenolic content of the beetroot juice

Nitrate and nitrite concentrations were measured in duplicate in samples from six separate bottles of Beet It® organic beetroot juice with lemon by GC-MS as isotope dilution after derivatisation with 2,3,4,5,6-pentafluorobenzyl bromide (PFB-Br) according to Tsikas^(^^18^^)^ with minor modifications. Briefly, known concentrations of [^15^N]nitrate and [^15^N]nitrite as internal standards were added to the beetroot juice. Then 200 µl spiked sample, 800 µl acetone and 20 µl PFB-Br were mixed and incubated for 20 min at 50°C in a sealed tube, after which the acetone was evaporated under an N_2_ stream. A quantity of 2 ml toluene and 1 ml water was added and shaken for 1 min. After phase separation, the upper (toluene) phase was analysed on a GC-MS (Shimadzu Corporation) with an Optima 17 column (15 m, 0·25 mm internal diameter, 0·25 µm film thickness), using negative-ion chemical ionisation, splitless mode, He (70 kPa) as column carrier, methane (200 Pa) as reagent. Initial column temperature was 70°C, held for 1 min, then increased 30°C/min to 280°C, with electron energy 230 eV and electron current 300 µA, ion source 180°C, interface 280°C and injector 200°C. [^15^N] and [^14^N]nitrate were measured at *m*/*z* 63 and 62, respectively, with retention time (RT) 3·2 min, [^15^N] and [^14^N]nitrite at *m*/*z* 47 and 46, respectively, with RT 3·4 min.

Betalains and phenolic compounds were measured using the method of Nemzer *et al.*^(^^19^^)^ on a Shimadzu Prominence HPLC with a Luna C-18 analytical column (25 cm, 3 mm internal diameter, 5 µm particle size; Phenomenex) and an SPD-M20A diode array detector (Shimadzu). Solvents were B, acetonitrile; and A, 2 % formic acid in water. The gradient was 3 % B at 0 min, 16 % B at 17 min, 50 % B at 30 min, 100 % B at 32–35 min and 3 % B at 35–45 min with 0·5 ml/min flow rate, 35°C column temperature and 10 µl injection volume. Peaks were recorded at 538 nm (betanins), 480 nm (betaxanthins and neobetanin), 505 nm (decarboxylated betanins) and 320 nm (phenolic compounds). Phenolic compounds were quantified at 320 nm rather than at the maximum wavelength for each compound; this was for practical reasons since most of them were not specifically identified, and this is a commonly used wavelength to measure phenolic compounds. Quantification of betanin, isobetanin, 17-decarboxyisobetanin and neobetanin was done by comparison with standards isolated by semi-preparative HPLC from the beetroot juice using the same equipment and solvents, on a Develosil ODS-HG-5 HPLC column (RP-18, 250 × 20 mm internal diameter), 5 µm particle size. The gradient was 3 % B at 0 min, 16 % B at 20 min, 50 % B at 40 min, 100 % B at 47 min until 60 min, with 3 ml/min flow rate, 35°C column temperature and 400 µl injection volume. Quantification of flavonoids and phenolic acids was done by comparison with authentic standards of rutin and chlorogenic acid, respectively, measured at 320 nm. In all, four peaks had UV spectra that matched typical flavonoid spectra and eighteen peaks were similar to phenolic acids.

### Subjects and study design

A group of sixteen healthy adults (six male and ten female) were recruited by poster for the present study. Subject characteristics are provided in Table 1. The present study was conducted according to the guidelines laid down in the Declaration of Helsinki and all procedures involving human subjects/patients were approved by the Oxford Brookes University Ethics Committee (110553). Written informed consent was obtained from all subjects. Participants were asked to visit the laboratory on three separate occasions. Before the first visit each participant was asked to complete a confidential health questionnaire pertaining to their medical history, a habitual physical activity questionnaire, and a modified FFQ designed to provide an estimate of their habitual polyphenol intake. Each visit was separated by no less than 48 h. At the start of each visit anthropometric measurements were taken and adherence to a 12 h fast, abstinence from caffeine, alcohol and intense physical activity as well as adequate hydration were assured verbally. Participants were excluded if they had type 2 diabetes or a fasting blood glucose measure of >6·1 mmol/l. Participants provided two baseline finger capillary samples separated by 5 min before consuming the test beverage. In a randomised, single-blind, cross-over design, three test beverages were provided to each subject.

**Table 1.** Participant characteristics (Mean values and standard deviations)

	Mean	sd
Age (years)	27	5
Height (cm)	172·4	8·6
Weight (kg)	69·2	11·7
BMI (kg/m^2^)	23·3	2·8
Fat (%)	23·0	7·0
Physical activity index		
Work	2·44	0·25
Sport	3·71	2·06
Leisure	3·19	0·49
Polyphenol intake (g/d)	1·93	0·59

### Study protocol

The first beverage (BEET) consisted of 225 ml Beet It® organic beetroot juice with lemon (<2 %) (James White Drinks Ltd) providing a total of 50 g available CHO. The second beverage (MCON) consisted of a matched control drink (225 ml) containing sucrose, fructose, glucose, pea protein isolate, inulin and sodium chloride providing a total of 50 g available CHO (the precise composition of BEET and MCON are shown in Table 2). The third beverage (GLUC) contained 50 g available CHO as glucose (225 ml). On each test day postprandial finger-prick blood samples were taken at 5, 15, 30, 45, 60, 90, 120 and 150 min (time points T5, T15, T30, T45, T60, T90, T120 and T150, respectively) to measure both blood glucose (5 µl) and plasma insulin (300 µl) using a Unistik 3 single-use lancing device (Owen Mumford). Glucose concentration was measured using a Hemocue Glucose 201+ analyser (HemoCue Limited) calibrated to plasma equivalent glucose concentrations. For each meal the incremental AUC (iAUC) was calculated geometrically ignoring the area below the baseline^(^^20^^)^. Plasma samples were analysed for insulin using a radioimmunoassay on an automated immunoanalyser (Cobas E411; Roche Diagnostics). Plasma was separated from whole blood by centrifugation (4000 rpm for 10 min) and stored at –40°C for no more than 1 month. The procedures used to measure the glucose and insulin responses were adapted from those described by Brouns *et al.*^(^^21^^)^ and Wolever^(^^22^^)^ and were in line with the recommendations of the FAO/WHO^(^^20^^)^.

**Table 2.** Nutrient composition of the beetroot juice beverage (BEET), matched control beverage (MCON) and glucose control beverage (GLUC)

Nutrient	BEET (per 225 ml)*	MCON (per 225 ml)	GLUC (per 225 ml)
Energy			
kJ	931	943	941
kcal	223	225	225
Reduced N (g)	0·91	0·91	–
Fat (g)	0·00	0·08	–
Carbohydrate (g)	50·4	50·4	50·0
Of which sucrose (g)	39·35	39·35	–
Of which glucose (g)	4·77	4·77	50·0
Of which fructose (g)	5·13	5·13	–
Fibre	1·13	1·13	–
Na (mg)	117	117	–

* Data from Eurofins Testing Ltd (unpublished results) on the carbohydrate composition of Beet It® organic beetroot juice with lemon.

FFQ were analysed using the Phenol-Explorer database^(^^23^^)^ to estimate habitual polyphenol intake. The Phenol-Explorer database lists food polyphenol content according to a number of common methods. In this instance the values obtained by the Folin–Ciocalteu method were used, as these are available for the highest number of foods, allowing for the best representation of habitual intake for this type of enquiry. Habitual polyphenol intake (g/d) is displayed in Table 1. Habitual physical activity was assessed using the physical activity index proposed by Baecke *et al.*^(^^24^^)^. Based on a scale, proportionate to the levels of physical exertion required to perform the activity, each question in a standard questionnaire is given a value between 1 and 5, with the exception of sport and exercise which is calculated as a product of the average energy used for the activity (MJ), intensity of performance and the time spent performing. Physical activity is displayed in Table 1.

### Insulin sensitivity

An index of insulin sensitivity (S_I_) was calculated using the oral glucose minimal model reported by Burattini *et al.*^(^^25^^)^ in their investigation of insulin action and secretion that used a model based on the oral glucose tolerance test. It is an amalgamation of the classic minimal model of glucose kinetics coupled with an equation describing the rate of appearance of glucose into the circulation^(^^26^^)^. The following equation describes the model:
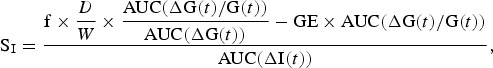
where AUC denotes the AUC of the quantities in parentheses during the total time course (*t*) 0–(*t*) 150, and ΔG(*t*) and ΔI(*t*) are glycaemia and insulinaemia above fasting values. GE is glucose effectiveness and f is the fraction of glucose appearing in the systemic circulation; values of 3·7 × 10^−2^ min/dl per kg and 0·87, respectively, are used in accordance with those reported by Burattini *et al.*^(^^25^^)^. 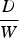 is the dose of glucose per kg body weight and is calculated individually for each subject.

### Statistical analyses

A mixed-model ANOVA with two factors (‘meal’ and ‘time’) was conducted but the assumed covariance matrix was discovered to be significantly different from the observed matrix. Therefore, comparisons of the data were made by repeated-measures ANOVA for glucose and insulin at each time point. Main effects were compared for the within-subjects factor ‘meal’ and corrected with a Bonferroni adjustment. Both iAUC and segmental AUC (sAUC) were also calculated and compared using repeated-measures ANOVA. Statistical power was calculated in consultation with the statistics department at Oxford Brookes University and is based on the mean and standard deviation of pilot data using similar conditions and participants. A minimum detectable difference of 10 % was chosen for insulin, as the spread of data from T0 to T150 is quite large and insulin is the primary outcome measure. Power was calculated using an online calculator for cross-over studies whereby the outcome is a measurement. The results showed that a power of 0·9 would be achieved at a significance level of *P* < 0·05 using sixteen participants.

## Results

### Phytochemical composition

Average nitrate concentration was 4·40 g/l, ranging from 3·03 to 5·24 g/l in six individual bottles, with an average relative sd within samples of 2·5 %. Average nitrite concentration was 2·34 mg/l, ranging from 2·23 to 2·55 mg/l in individual bottles, with an average relative standard deviation within samples of 2·0 %. There was no correlation between nitrate and nitrite concentrations when comparing individual bottles (*R*^2^ 0·147; *P* = 0·781). Values for the sum of betalains, betaxanthins, flavonoids, phenolic acids and some individual betalains are shown in Table 3. The beetroot juice was a particularly rich source of the orange/yellow pigment neobetanin (1263 mg per 225 ml) and contained a total of 129 mg polyphenolic compounds per 225 ml. Analysis of the CHO content of the beetroot juice showed that the composition was 90 % sucrose, 5 % glucose, 4 % fructose with minor amounts of maltose and lactose (<0·1 g per 100 g). Crude protein content was 2·53 g per 100 g, and total fibre was <0·5 g per 100 g. The value for neobetanin was adjusted for a low recovery (47·5 %) measured by quantification of a purified sample with analytical HPLC and compared with the spectrophotometric value. The recovery was even less during semi-preparative HPLC.

**Table 3.** Phytochemical composition of the beetroot juice beverage (Mean values with their standard errors (*n* 6), and relative standard deviations)

	Concentration (mg/l)		
Compound	Mean	sem	Relative sd (%)
Sum of betaxanthins (yellow native pigments)	14·6	1·1	18
Sum of betanins (purple native pigments)	215	23	26
Sum of betanin degradation products	5975	68	2·8
Sum of flavonoids (rutin equivalents)	323·14	0·69	0·52
Sum of phenolic acids (chlorogenic acid equivalents)	248·2	4·6	4·5
Betanin	83·7	8·0	23
Isobetanin	131	15	28
17-Decarboxy-isobetanin	22·56	0·40	4·3
Neobetanin	5617	65	2·9

### Glycaemic response

**Fig. 1.**
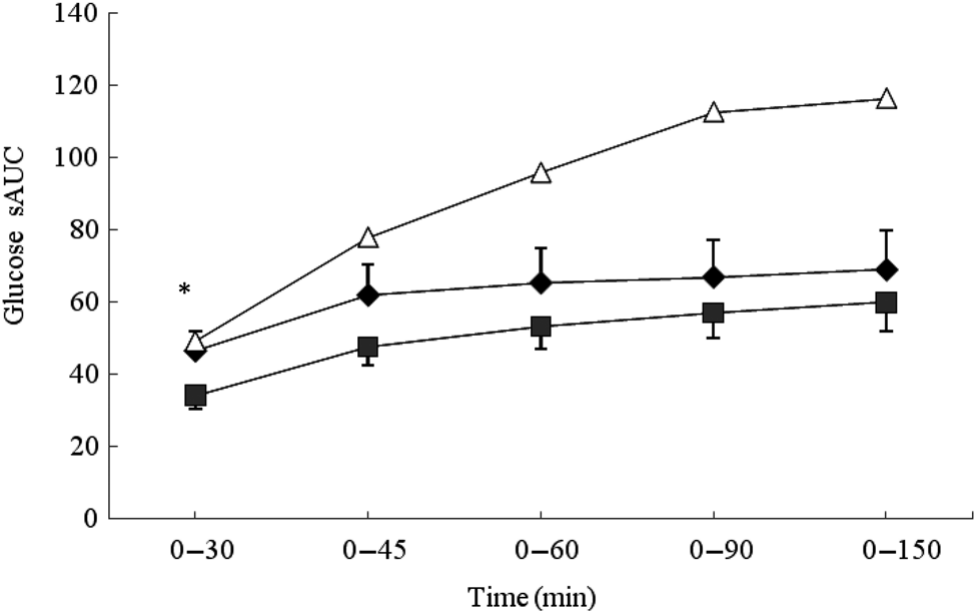
Segmental areas under the blood glucose response curve (sAUC). Values are means (*n* 16), with standard deviations represented by vertical bars. For clarity, negative error bars are shown for the beetroot beverage (BEET; ■) and positive error bars are displayed for the matched control beverage (MCON; ♦). Error bars are not displayed for the glucose beverage (GLUC; Δ) but were comparable in magnitude. * Mean value for BEET was significantly different from that for MCON (*P* < 0·05).

No significant differences were observed between the MCON and BEET beverages during the total glycaemic response (iAUC). Both the MCON and BEET conditions produced a significantly lower glycaemic response than the GLUC condition (*P* < 0·05), as expected (Table 4). Analysis of the AUC by segment (sAUC) revealed a significantly lower glycaemic response for BEET compared with MCON in the 0–30 min segment (*P* < 0·05) (Fig. 1). BEET was also significantly lower than GLUC in the 0–30 min and 0–45 min segments. In the 0–60 min segment BEET remained significantly lower than GLUC (*P* < 0·05). Both MCON and BEET were significantly lower than GLUC (*P* < 0·05) in the 0–90 min segment. Data were also analysed by time point. Significant differences between the experimental conditions are shown in Table 4. BEET was significantly lower (*P* < 0·05) than MCON at T5. BEET was significantly lower (*P* < 0·05) than GLUC at T30, and BEET and MCON were both significantly lower than GLUC at T45, T60 and T90.

**Table 4.** Incremental AUC analysis for glucose and insulin (Mean values and standard deviations, *n* 16)

	MCON		BEET		GLUC	
Time (min)	Mean	sd	Mean	sd	Mean	sd
Glycaemic response						
5	2·7	1·9	1·4	1·3*	1·3	1·5
15	15·4	7·0	10·7	5·7	14·1	7·4
30	28·3	15·9	22·0	10·0†	33·9	12·8
45	15·5†	14·6	13·5	10·2†	28·7	17·8*
60	3·4†	8·3	5·6	7·2†	17·9	13·7*
90	1·6†	3·9	3·9	6·7†	16·8	16·3*
120	0·7	1·9	1·6	3·7†	3·6	5·7
150	1·4	2·3	1·3	2·5†	0·2	0·5
Total	69	44	60	33	116	60
Insulin response						
5	63	57	35	27	37	34
15	354†	207	227	144*	222	133
30	635†	334	444	346	465	349
45	397	292	313	268	408	369
60	166	116	173	148	253	227
90	82	108	170	177	216	289
120	8	21	43	94*	55	119
150	5	19	4	14	5	13
Total	1694	842	1409	959	1661	1109

MCON, matched control beverage; BEET, beetroot beverage; GLUC, glucose beverage.

* Mean value was significantly different from that of the MCON condition (*P* < 0·05).

† Mean value was significantly different from that of the GLUC condition (*P* < 0·05).

### Insulin response

**Fig. 2.**
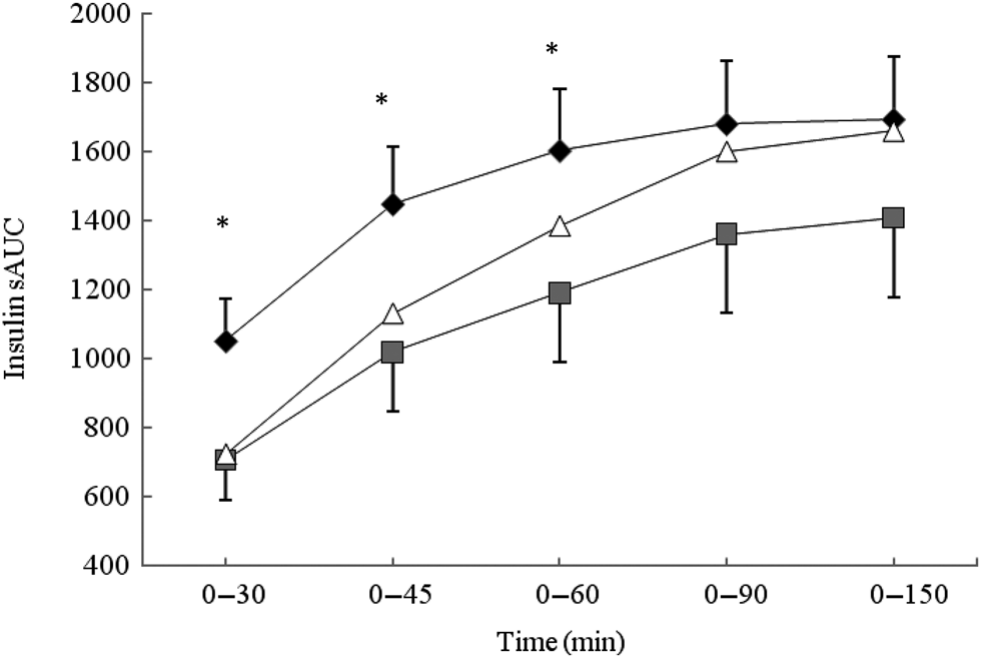
Segmental areas under the blood insulin response curve (sAUC). Values are means (*n* 16), with standard deviations represented by vertical bars. For clarity, negative error bars are shown for the beetroot beverage (BEET; ■) and positive error bars are displayed for the matched control beverage (MCON; ♦). Error bars are not displayed for the glucose beverage (GLUC; Δ) but were comparable in magnitude. * Mean value for BEET was significantly different from that for MCON (*P* < 0·05).

iAUC analysis revealed no significant difference between the three conditions for insulin response (Table 4). The data were separated into the 0–30, 0–45, 0–60 and 0–90 min segments and further analysed (Fig. 2). The BEET condition elicited a significantly lower insulin response in the 0–30, 0–45 and 0–60 min segments (*P* < 0·05 for each) compared with MCON. The data were also analysed by time point (Table 4). A non-significant, lower insulin response was observed for BEET compared with MCON at T5 (*P* = 0·08), which reached significance at T15 (*P* < 0·05) and remained evident, although not significant, at T30 (*P* = 0·09). The BEET condition was shown to elicit a significantly higher insulin response than MCON at T120 (*P* < 0·05), although the actual values are relatively small.

### Physical activity and polyphenol consumption

The mean physical activity index for this cohort was 3·11 (sd 0·75). Individually, the work index was 2·44 (sd 0·25), the sport index was 3·71 (sd 2·06) and the leisure index was 3·19 (sd 0·49). These values indicate a recreationally active cohort. Polyphenol intake for this cohort was 1925 (sd 592) mg/d with a range 853–2807 mg/d. This suggests a cohort with a relatively high habitual intake of polyphenols.

### Insulin sensitivity

Insulin sensitivity was calculated according the minimal model proposed by Caumo *et al.*^(^^26^^)^. The data were log transformed to reduce the impact of high inter-individual variability in insulin response. No significant differences were observed between the conditions. Insulin sensitivity was 1·05 × 10^−5^ dl/kg per min per pmol/l (95 % CI 0·55, 2·01) for MCON compared with 1·68 × 10^−5^ dl/kg per min per pmol/l (95 % CI 0·86, 3·29) for the BEET condition. Insulin sensitivity in the GLUC condition was 2·10 × 10^−5^ dl/kg per min per pmol/l (95 % CI 1·08, 4·06).

## Discussion

The aim of the present study was to investigate the effects of a beetroot juice on postprandial glucose and insulin concentrations as well as insulin sensitivity and characterise the phytochemicals of this juice. There are two possible confounders of these results that are hereby considered. Acids have been shown to reduce postprandial glucose and insulin concentrations^(^^27^^,^^28^^)^. There is a small amount of lemon juice present in the beetroot beverage (<2 %); Leeman *et al.*^(^^27^^)^ showed that acids were required in gram quantities to observe a significant reduction in postprandial glycaemia. The possible confounding of these results by the lemon juice in this beverage may therefore be considered to be negligible. Beetroot juice contains a number of bioactive components including betaxanthins, betanins, flavonoids, phenolic acids^(^^15^^)^ and betanin degradation products produced by thermal processing (Table 3). The concentration of betaxanthin was relatively low (3·29 mg per 225 ml), as was the concentration of intact betanins (48·4 mg per 225 ml). The betanins were divided into 18·8 mg betanin and 29·5 mg isobetanin. Both flavonoids and phenolic acids were present in moderate concentrations (72·7 mg per 225 ml and 55·9 mg per 225 ml, respectively). The most abundant compounds in the beetroot juice were betanin degradation products formed, presumably, by thermal processing techniques. These were divided into two predominant compounds: 17-decarboxy-isobetanin (5·08 mg per 225 ml); and the orange/yellow pigment; neobetanin (1264 mg per 225 ml). The much higher CV (among bottles) for the betalain pigments than for the non-betalain types of phenolic compounds indicates that some of the pigment degradation took place after the juice was distributed into the containers, while the non-betalain compounds did not degrade. The contents of nitrate and nitrite were within the ranges previously found for processed beetroot products^(^^29^^)^. The nitrite levels were relatively low, indicating that little or no bacterial denitrification had occurred during processing and storage.

There was a similar glycaemic response across the three conditions with the exception of a significantly lower glycaemic response at 5 min (*P* < 0·05), which persisted, although not significantly, across the proceeding minutes, sufficient to cause a significantly lower glycaemic response (*P* < 0·05) to be detected in the 0–30 min sAUC for BEET compared with MCON. The moderately attenuated glycaemic response was accompanied by a similarly attenuated early-phase insulin response. A significantly lower insulin response was detected at 15 min for BEET compared with MCON, with further lower responses measured at 5 and 30 min that did not reach significance (*P* = 0·08 and *P* = 0·09, respectively). These differences were sufficient to cause significant differences to also be observed in the sAUC segments between 0–30, 0–45 and 0–60 min for BEET compared with MCON. These results suggest, although not conclusively, that bioactive compounds within beetroot juice may have the potential to help control postprandial glycaemia. Participants were asked to refrain from unusual eating habits, but some participants may still have consumed large quantities of phytochemicals or particularly high quantities of CHO with their evening meal. This is unlikely to have had a significant impact upon the results since the half-life of polyphenols and betalains is reported to be approximately 5 h in the blood^(^^30^^,^^31^^)^.

Törrönen *et al.*^(^^32^^)^ fed fourteen healthy participants a blackcurrant juice beverage containing 50 g sucrose fortified with crowberry powder, which doubled the polyphenol content compared with the placebo beverage. The crowberry powder was a particularly rich source of anthocyanins, a group within the flavonoid family, and the test beverage provided 293 mg polyphenols per 100 ml. The postprandial glycaemic response in the study by Törrönen *et al.*^(^^32^^)^ followed a similar pattern to the present study with no significant differences overall, but a larger (although not significant) glucose and insulin response observed in the early phase (up to 30 min) for the control condition compared with the test condition. Törrönen *et al.*^(^^32^^)^ assigned their results to the higher polyphenol content of the test juice, and suggested reduced digestion of sucrose and/or a slower release of glucose in the gut as the likely mechanism(s). The addition of berries, rich in polyphenols, to a sucrose load has previously been shown to attenuate the postprandial glucose and insulin responses in adults^(^^33^^–^^35^^)^. A number of other studies have also investigated the effect of polyphenol-rich interventions on glucose and/or insulin response in healthy subjects. Overall, a high proportion of these studies show a delayed rise in glucose and/or insulin in the early phase of the postprandial response, which reaches significance in some but not all studies. These studies are summarised in Table 5^(^^7^^,^^32^^–^^45^^)^. Some studies have analysed blood taken from venous samples whilst others (including the present study) have utilised capillary sampling. No difference has been observed between capillary and venous sampling, although capillary sampling has previously been suggested to be more accurate^(^^21^^)^.

**Table 5.** Review of studies investigating the effects of phytochemicals on postprandial glucose and insulin

Study	Subjects	Treatment	Compound	Delayed response in early phase for glucose or insulin?	Significance
Cao *et al.*^(^^36^^)^	Eight healthy elderly women	240 g strawberries, 294 g spinach, 300 ml red wine or 1250 mg vitamin C *v.* matched control	Various including anthocyanins (strawberries)	No	N/A
Johnston *et al.*^(^^7^^)^	Nine healthy adults	400 ml clear or cloudy apple juice *v.* CHO-matched control	Chlorogenic acid	Yes (glucose) at 15, 30 min (clear) and 15 min (cloudy)	*P* < 0·05
Kay & Holub^(^^37^^)^	Eight healthy men	100 g freeze-dried wild blueberry powder in 500 ml water + high-fat meal *v.* matched placebo control	Anthocyanins	No	N/A
Bryans *et al.*^(^^38^^)^	Sixteen healthy adults; 4 m, 12 f	1 or 3 g black tea + 75 g glucose in 150 ml water *v.* CHO-matched control	Tea polyphenols: flavan-3-ols, catechins	No. Late-phase reduction in glucose	N/A
Wilson *et al.*^(^^39^^)^	187 healthy adults	High- or low-energy 240 ml cranberry juice + 80 mg vitamin C *v.* matched controls	Proanthocyanidins	Yes (glucose) at 30 min Yes (insulin) up to 60 min	No
Hlebowicz *et al.*^(^^40^^)^	Fifteen healthy adults; 9 m, 6 f	300 g rice pudding + 1 or 3 g cinnamon	Methyl hydroxychalcone	Yes (both) up to 60 min	No (glucose) Yes (insulin)
Josic *et al.*^(^^41^^)^	Fourteen healthy adults; 7 m, 7 f	300 ml green tea together with white bread and sliced turkey *v.* CHO-matched control	Flavan-3-ols, catechins	Yes (glucose) up to 60 min	No
Lehtonen *et al.*^(^^42^^)^	Ten healthy men	Sea buckthorn crushed berries + yogurt + 50 g glucose *v.* matched control	Flavonols	Yes (insulin) at 30 min	*P* < 0·05
Törrönen *et al.*^(^^33^^)^	Twelve healthy adults; 1 m, 11 f	150 g berry purée (blackcurrants, cranberries, strawberries, bilberries) + 35 g sucrose (800 mg total polyphenols) *v.* sucrose + CHO-matched control	Anthocyanins	Yes (glucose) at 15 and 30 min	*P* < 0·05 (15 min), *P* < 0·01 (30 min)
Clegg *et al.*^(^^43^^)^	Twelve healthy adults; 3 m, 9 f	100 g blueberries or raspberries in a pancake meal *v.* CHO-matched control and glucose	Anthocyanins	No	N/A
Wootton-Beard & Ryan^(^^45^^)^	Seventeen healthy adults; 7 m, 10 f	70 ml beetroot juice + white bread *v.* CHO-matched control	Betalains	Yes (insulin) at 15 min	*P* < 0·05
Törrönen *et al.*^(^^32^^)^	Fourteen healthy adults; 3 m, 11 f	300 ml blackcurrant juice or blackcurrant juice fortified with crowberry powder (mainly anthocyanins 89 mg/100 g *v.* 178 mg per 100 g)	Anthocyanins	Yes (glucose) at 30 min Yes (insulin) up to 45 min	No
Törrönen *et al.*^(^^34^^)^	Fifteen healthy adults	150 g berry purée + 35 g sucrose *v.* sucrose + CHO-matched control	Anthocyanins	Yes (glucose) up to 45 min Yes (insulin) at 15 min	*P* < 0·05 (15 min), *P* < 0·01 (15 min)
Törrönen *et al.*^(^^35^^)^	Twenty healthy women	150 g blackcurrant and lingonberry purée or nectar + 35 g sucrose *v.* CHO-matched control	Anthocyanins	Yes (glucose) up to 30 min Yes (insulin) up to 30 min	No
Linderborg *et al.*^(^^44^^)^	Ten healthy men	40 g lingonberry powder in yoghurt + 50 g glucose *v.* CHO-matched control – lingonberry	Flavonol glycosides	Yes (glucose) same as berries Yes (insulin) up to 60 min	No No

N/A, not applicable; CHO, carbohydrate; m, male; f, female.

Previous studies have reported polyphenol intakes of 300–800 mg as sufficient to attenuate postprandial glycaemia when consumed alongside CHO^(^^32^^,^^34^^)^. The beetroot juice beverage in the present study provided a total of 129 mg of polyphenols together with 1393 mg of betanins and betanin degradation products, in particular 1263 mg neobetanin. The present study thus suggests that betanins (particularly the degradation product neobetanin) and/or nitrate may have comparable effects to phenolic compounds, since the content of flavonoids and phenolic acids contained in beetroot juice is too low to fully account for the observed effects. Neobetanin was identified in the 1980s as the orange/yellow pigment 5-*O*-β-d-glucopyranosylneobetanidin isolated from red beetroot^(^^46^^–^^48^^)^.

As has been discussed previously, the potential beneficial effects of phytochemicals on the control of postprandial glycaemia may be due to a reduced digestion of glucose, reduced absorption of glucose, stimulation of insulin release or alterations in insulin signalling/sensitivity. The reduced glucose response in the 0–30 min period following consumption of the beetroot beverage and the persistently reduced insulin response over the first 60 min also observed following ingestion of the beetroot beverage in the present study suggest that either glucose uptake is blunted, resulting in reduced insulin secretion, or that less insulin is required to restore glucose homeostasis resulting in a reduction in circulating insulin. There is not sufficient evidence presented in the present study to eliminate any potential mechanism, nor are the differences observed in the responses sufficient to confirm any of the potential mechanisms, although there is a suggested increase in insulin sensitivity. The insulin sensitivity model that was applied to the data did not reveal any significant differences between the three conditions, which may be largely due to the apparent inter-individual variability of human glycaemic response data. The beetroot condition did result in a higher insulin sensitivity score (1·68 (95 % CI 0·86, 3·29) × 10^−5^ dl/kg per min per pmol/l) than the matched control condition (1·05 (95 % CI 0·55, 2·01) × 10^−5^ dl/kg per min per pmol/l), which did not approach significance (*P* = 0·24).

The high concentration of betalains found in the beetroot juice, particularly betanin degradation products such as neobetanin, suggests that they are the most likely compounds to be responsible for any of the alterations observed. Betalains bear certain structural similarities to the anthocyanin group of polyphenolic compounds, a group which has also been shown to alter postprandial glycaemia^(^^32^^)^. Each species of plants contains either betalains or anthocyanins, since the two groups of compounds play exactly the same role in the plant^(^^49^^)^. It would therefore not be surprising if these compounds also have similar effects on mammalian physiology.

A review of ninety-seven bioavailability studies showed that ingested polyphenolic compounds reach the low micromolar range in plasma^(^^30^^)^. These concentrations were observed for flavanols and catechins, whilst anthocyanins reached only high nanomolar concentrations. There are no studies to date assessing the bioavailability or metabolism of betalain degradation products in humans. Tesoriere *et al.*^(^^50^^)^ examined the absorption, excretion and distribution of native betalains from cactus pear fruit in human LDL. Tesoriere *et al.*^(^^50^^)^ showed that betanin contained in cactus pear fruit reached a maximum plasma concentration of 0·2 µmol/l after approximately 3 h following an intake of 16 mg, which indicates that betalains reach the plasma in relatively high concentrations compared with anthocyanins. Betalains have a short half-life in the plasma and erythrocytes (about 5 h)^(^^31^^)^, which may limit their potential function to processes lasting only a short period such as glucose digestion and absorption. For this potential effect to be prolonged, the desired compounds would need to be ingested in sufficient quantity, at each eating episode containing CHO. The effect of nitrate in this phenomenon cannot be ruled out since polyphenols can mimic nitrate by enhancing the endogenous production of NO^(^^51^^)^. However, the opposite effect of nitrate mimicking polyphenols has yet to be studied.

### Conclusions

This is the first study to investigate the effects of beetroot juice on postprandial glycaemia. The study showed that beetroot juice elicited a significant suppression of postprandial glycaemia in the 0–30 min sAUC (*P* < 0·05) and postprandial insulinaemia in the 0–30, 0–45 and 0–60 sAUC (*P* < 0·05) when compared with a control beverage matched for CHO content, which was comparable with that observed using berries in other investigations. HPLC analysis of the beetroot juice revealed that the predominant secondary metabolite (excluding nitrate) in this product is the yellow/orange pigment neobetanin, derived from betanin, as well as significant, but much smaller, amounts of other betalains and polyphenolic compounds. Neobetanin's size and chemical properties (for example, conjugated system) are similar to those of flavonoid polyphenolics such as anthocyanins and it is suggested, therefore, that neobetanin probably contributes to the observed effect, which is similar to effects previously reported for polyphenolic compounds. Insulin sensitivity models applied to the data suggested that insulin sensitivity in the tested cohorts was non-significantly increased with beetroot juice compared with control.

The potential for bioactive phytochemicals to modify substrate metabolism in the postprandial state is a developing area of interest. Reports by Murase *et al.*^(^^52^^)^ on coffee polyphenols (caffeic acid derivatives), Tiwari *et al.*^(^^53^^)^ on the effects of Indian vegetable juices and Linderborg *et al.*^(^^44^^)^ on sea buckthorn have all demonstrated significant effects on glucose and insulin responses. Hyperglycaemia, hyperinsulinaemia and hyperlipidaemia are crucial in the development of CVD, type 2 diabetes and the metabolic syndrome, and whether or not they are able to be modified by dietary components is of significant ongoing interest.
